# Using a coupled dynamic factor – random forest analysis (DFRFA) to reveal drivers of spatiotemporal heterogeneity in the semi-arid regions of southern Africa

**DOI:** 10.1371/journal.pone.0208400

**Published:** 2018-12-14

**Authors:** Jane Southworth, Erin Bunting, Likai Zhu, Sadie J. Ryan, Hannah V. Herrero, Peter Waylen, Rafael Muñoz-Carpena, Miguel A. Campo-Bescós, David Kaplan

**Affiliations:** 1 Geography Department, University of Florida, Gainesville, Florida, United States of America; 2 Department of Geography, Environment, and Spatial Sciences; Remote Sensing and GIS Research and Outreach Services, Michigan State University, East Lansing, Michigan, United States of America; 3 Silvis Lab, University of Wisconsin, Madison, Wisconsin, United States of America; 4 Emerging Pathogens Institute, University of Florida, Gainesville, Florida, United States of America; 5 College of Life Sciences, University of KwaZulu Natal, Pietermaritzburg, South Africa; 6 Agricultural and Biological Engineering Department, University of Florida, Gainesville, Florida, United States of America; 7 Department of Projects and Rural Engineering, Public University of Navarre, Pamplona, Spain; 8 Department of Environmental Engineering Sciences, University of Florida, Gainesville, Florida, United States of America; University of Oregon, UNITED STATES

## Abstract

Understanding the drivers of large-scale vegetation change is critical to managing landscapes and key to predicting how projected climate and land use changes will affect regional vegetation patterns. This study aimed to improve our understanding of the role, magnitude, and spatial distribution of the key environmental and socioeconomic factors driving vegetation change in a southern African savanna. This research was conducted across the Kwando, Okavango and Zambezi catchments of southern Africa (Angola, Namibia, Botswana and Zambia) and explored vegetation cover change across the region from 2001–2010. A novel coupled analysis was applied to model the dynamic biophysical factors then to determine the discrete / social drivers of spatial heterogeneity on vegetation. Previous research applied Dynamic Factor Analysis (DFA), a multivariate times series dimension reduction technique, to ten years of monthly remotely sensed vegetation data (MODIS-derived normalized difference vegetation index, NDVI), and a suite of time-series (monthly) environmental covariates: precipitation, mean, minimum and maximum air temperature, soil moisture, relative humidity, fire and potential evapotranspiration. This initial research was performed at a regional scale to develop meso-scale models explaining mean regional NDVI patterns. The regional DFA predictions were compared to the fine-scale MODIS time series using Kendall’s Tau and Sen’s Slope to identify pixels where the DFA model we had developed, under or over predicted NDVI. Once identified, a Random Forest (RF) analysis using a series of static social and physical variables was applied to explain these remaining areas of under- and over- prediction to fully explore the drivers of heterogeneity in this savanna system. The RF analysis revealed the importance of protected areas, elevation, soil type, locations of higher population, roads, and settlements, in explaining fine scale differences in vegetation biomass. While the previously applied DFA generated a model of environmental variables driving NDVI, the RF work developed here highlighted human influences dominating that signal. The combined DFRFA model approach explains almost 90% of the variance in NDVI across this landscape from 2001–2010. Our methodology presents a unique coupling of dynamic and static factor analyses, yielding novel insights into savanna heterogeneity, and providing a tool of great potential for researchers and managers alike.

## Introduction

Savanna landscapes encompass a fifth of the world’s ecosystems including regions in South America, Australia, and roughly 65% of Africa [1,2]. These landscapes, which represent the intermediate ecosystem between forests and grasslands, are of great ecological importance due to high biological productivity [3–5], their role in the global carbon cycle [6–9], and support of large human populations through natural resource use [10].

Structurally, savannas are defined as seasonal landscapes with a continuous herbaceous layer and discontinuous woody (tree and shrub) cover [1]. The structure and composition of savanna landscapes is prone to fluctuations as drivers of spatial heterogeneity change the system. Such drivers of spatial heterogeneity have long been studied to determine the degree and scale of impact to the system (eg. [11]). However, the cumulative effect of drivers of landscape change remains understudied, largely due to the complexity of such analyses. No single driver is completely responsible for changing an ecosystem’s land cover and biological function. Instead it is the combination of both biophysical and social drivers, which dictate vegetation greenness and overall productivity. Climate variability, soil moisture, fire, herbivory, soil nutrients, and human impacts are key drivers of spatial heterogeneity [11–15]. Previous research has illustrated the variation in biophysical drivers across this landscape [16], absent any social or static environmental factors at a regional scale. In order to develop landscape management policies and gauge the resilience of savanna landscapes, it is crucial to further our understanding of the role of these drivers at a more local (pixel-level) scale, and to study them in combination with one another.

Across savanna landscapes water availability is of critical importance and typically is the dominant driver of landscape change and overall biological function [1,11,16]. Numerous studies have assessed the impact of water availability and associated soil moisture on savanna vegetation showing that the role of water varies across different vegetation types and biomes [11,16]. Overall, the physiological process for which water, i.e. precipitation, drives vegetation productivity / greenness is quite simple. Rainfall stimulates green-up of vegetation, especially woody vegetation, and such water availability determines the duration of growth. The other environmental factors, such as herbivory, soil, and nutrients play a significant role across the landscape but typically at more localized scales [11].

Vegetation productivity is influenced by human use of the landscape. For the past several decades, the global push for conservation and sustainable biodiversity has focused on protectionism [17,18], effectively isolating PAs, and managing them as individual, distinct landscapes. However, recent research and management practices have expanded beyond protected area boundaries, to encompass larger scale ecological and anthropogenic processes in the overall landscape [17]. Smaller protected areas cannot maintain high levels of biodiversity at a sustainable level, and are usually developed without a buffering mechanism (buffering from surrounding human populations), resulting in much of the biodiversity remaining outside of protected areas and thus potentially more vulnerable [19].

In African savannas, as the human footprint on the landscape increases, the change in conservation approaches is particularly prominent. In recent years countries have come together to join formerly fragmented protected areas and their surrounding landscapes to form some of the largest transfrontier conservation zones (TCZs) in the world [17]. Development of TCZs is important given that most protected areas are too small to encompass the home ranges of majestic megafauna and the ecological processes that dictate landscape function [20,21]. One of the largest TCZs, the Kavango-Zambezi Transfrontier Conservation Zone (KZTCZ), was established in 2010, encompassing large portions of the savanna landscapes of southern Africa including part of our study area. As such, it is key for us to include the role of management and understand the impact of protected areas on the landscape.

Remotely sensed data provides the means to develop monitoring strategies for savanna landscapes, across varying spatial scales and at regular time steps. Traditionally, spectral indices such as the Normalized Difference Vegetation Index (NDVI) have been used as proxy measures of net primary productivity and greenness, but this index can also be used to study the underlying drivers of landscape composition and change [22–24]. What is key in studies of drivers of landscape composition, and sometimes lacking, is a statistical or modeling methodology that incorporates both continuous (e.g. time series) and discrete (e.g. boundaries, single time events) variables. Without a methodological framework that encompasses all drivers of spatial heterogeneity, interpretation of landscape processes and drivers of change is difficult. In this study, a novel coupled methodology was developed, to better understand drivers of landscape change across complex covarying systems, such as savanna landscapes. The coupled methodology involved an advanced time-series modeling of vegetation (Normalized Difference Vegetation Index) at a regional scale (DFA), based solely on biophysical variables, followed by a statistical test which assessed the impact of both anthropogenic and biophysical variables, at a pixel level (RF). We call this coupled methodology the DFRFA. Ultimately, this methodology provides a useful analytical tool for understanding landscape productivity and vegetation change, which incorporates both discrete and continuous data.

## Materials and methods

### Study area

**Fig 1:**
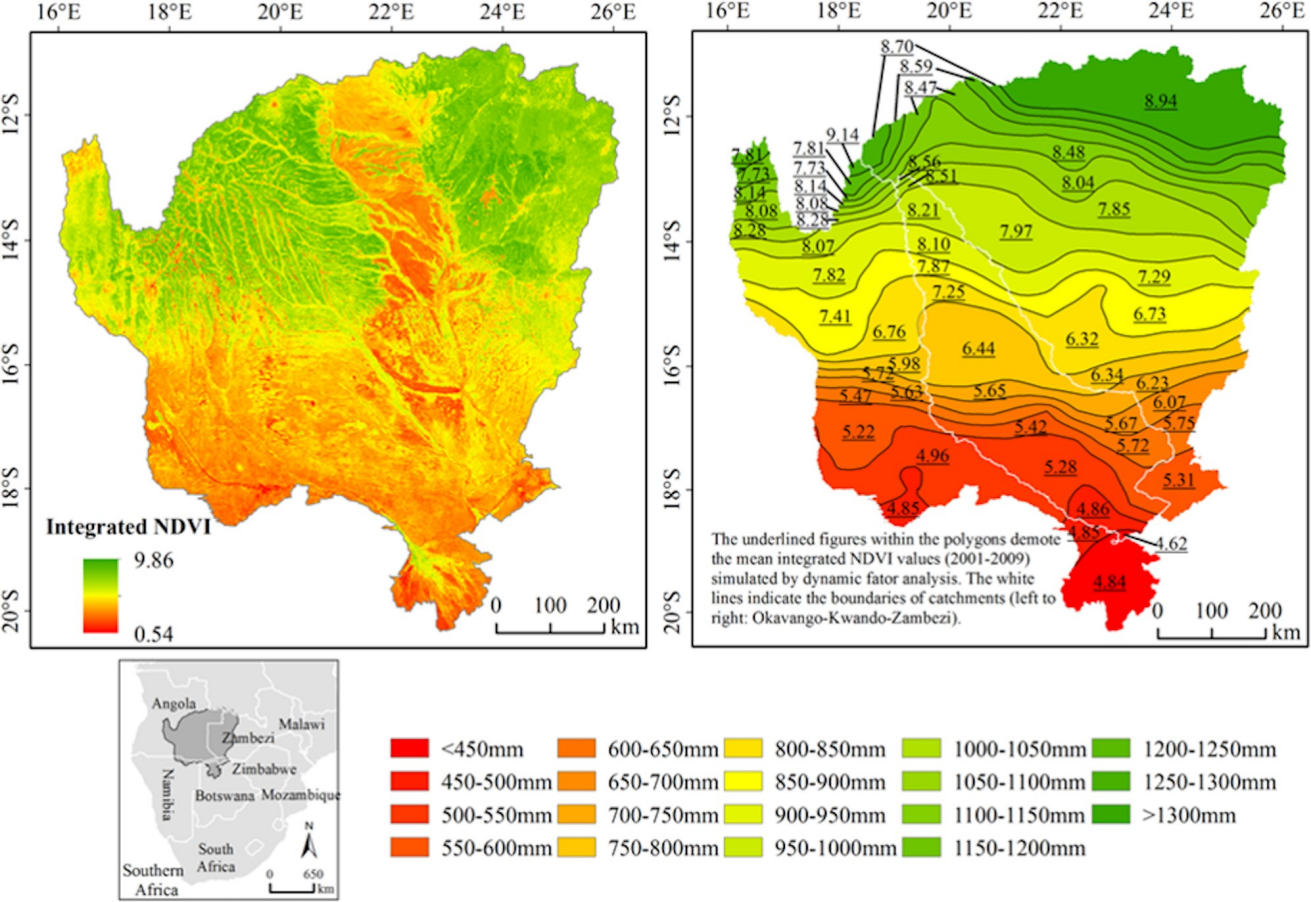
The geographic location of the study area. The upper left map shows the mean annual integrated NDVI for 2001–2010. The upper right map illustrates the 48 polygons associated with different precipitation intervals and the figures within the map shows the mean integrated annual NDVI values simulated by dynamic factor analysis at polygon level. The inset map shows how the study area is located in relation to the larger region of southern Africa.

Vast portions of sub-Saharan Africa are classified as arid to semi-arid, following their precipitation regimes. This study focuses on the arid to semi-arid landscapes across of the Okavango, Kwando, and Zambezi Catchments (Fig 1). These span Botswana, Namibia, Zambia, Zimbabwe, and Angola, covering more than 683,000 km^2^. The headwaters for all three catchments, and the majority of the catchment areas lie within Angola. Across the study area, a distinct seasonal phenological cycle is exhibited which, for the most part, is driven by the migration of the Inter Tropical Convergence Zone (ITCZ). The wet season traditionally runs from mid-September/October to Late April/May, with average annual precipitation during this time ranging from 1400 mm/yr to <400 mm/yr. The dry season occurs in the other months and is associated with large losses in soil moisture, lower water levels in standing water, and in some cases drought like conditions. Precipitation exhibits a steep north to south gradient characterized by low precipitation totals and higher interannual variability in the southern most regions and an increase in precipitation in the northern portions of the study area [25]. Extreme or long-term changes in the precipitation regime are related to the El Niño Southern Oscillation (ENSO) phase and other major ocean oscillations, such as the Indian Ocean Dipole (IOD) [26]. There is a long history of climatic shifts in the region, the most notable of which occurred in the late 1970’s, which correlates with global climate shifts, and a decrease in precipitation in southern Africa [27]. In addition to climatological patterns, there is a distinct human footprint, unique biological diversity, and differing landscape management policies. While less heavily populated than some regions of west and east Africa, there are many villages in the study area, almost all of which depend on the land and natural resource extraction / utilization for economic stability. Two of the main livelihood strategies in the region are agriculture and tourism, illustrating the coupled socio-ecological nature of the system [28].

The study region straddles the critical threshold of 650 mm Mean Annual Precipitation (MAP) [29]. Below this threshold, precipitation is found to be the dominant driver of vegetation change, as soil available moisture is limited and therefore plant growth is restricted and highly competitive. Above the 650 mm threshold, other factors such as herbivory and fires play a key role in spatial heterogeneity [29], as these areas received sufficient precipitation to support both herbaceous and woody plant growth. Our previous research [16] found this critical threshold to be closer to 850 mm but there is clearly a significant precipitation gradient, thresholds of change in drivers on the landscape, and significant social and political variation. As such, this is an ideal landscape for this study which looks to integrate biological and social/political/economic drivers of change across both time series based methods and discrete classifiers and utilizing both time-series and static landscape variables.

### Data

Both continuous environmental and discrete social and physical data are key drivers of vegetation biomass on the landscape. In this study, we used a novel two-step methodological approach (DFRFA) to explore details of key drivers of vegetation change (i.e. pattern and process). Specifically, a suite of both continuous and discrete variables types are used to predict and explain vegetation patterns across the study region from 2001–2010.

### Predicted NDVI values [NDVI_pred_]

In previous research [16] a Dynamic Factor Analysis (DFA) of biophysical time series covariates was used to model the drivers of regional Normalized Difference Vegetation Index (NDVI) in the landscape. DFA requires continuous data series, which is useful for analyzing NDVI. NDVI has a theoretical range from -1 to +1, but realistically across this landscape values could range from 0 (no vegetation) to +1 dense green leaf vegetation. Globally, NDVI is one of the most commonly used spectral indices employed due to the manipulation of the red and NIR spectral bands to highlight contrasting absorption and reflection patterns. The DFA model, fully detailed in [16], was used to predict NDVI for 48 polygons across our landscape (Fig 1). The polygons were based on the north-south precipitation gradient; specifically the long-term precipitation means divided into 50 mm intervals. Additionally, the polygons were divided by catchments (Fig 1).

The DFA model was developed from monthly time series of regional NDVI data and environmental covariates, which span 10 years from 2001–2010, comprising time series of precipitation, temperature, fire/thermal anomalies, soil moisture, relative humidity, and potential soil moisture. NDVI data was obtained from the Moderate Resolution Imaging Spectroradiometer (MODIS) via the MOD13A3 product. The gridded vegetation index was calculated from bi-directional surface reflectance that has been masked for water, clouds, heavy aerosols, and shadow (USGS). Four MODIS tiles cover the study area for this research project: H19V10, H19V11, H20V10, H20V11.

### Explanatory variables: Random forest analysis

**Fig 2:**
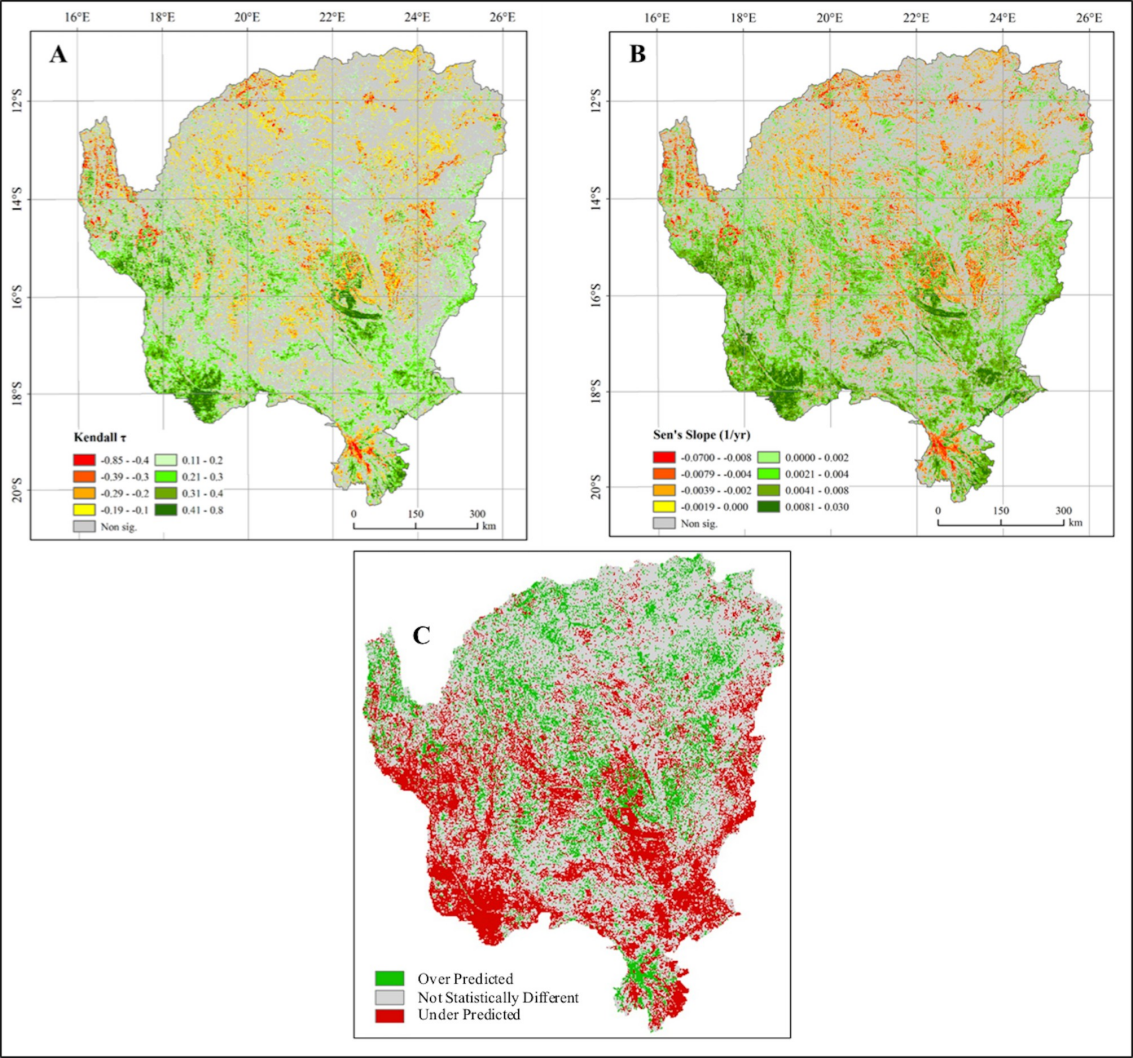
Spatial pattern of the relationship between the DFA NDVI prediction and the actual MODIS NDVI time series. (A) Kendall’s correlation coefficient (*τ*). The positive value indicates an increasing trend, whereas the negative value indicates a decreasing trend (at significant level of 0.1). (B) Spatial pattern of Sen’s slope of the OKZ catchment. The positive value indicates an increasing trend, whereas the negative value indicates a decreasing trend. (C) Simplified map of the model output illustrating areas where NDVI was under predicted in green and over predicted in red.

The vegetation in the semi-arid to arid savanna landscapes of southern Africa is in a constant state of flux at multiple spatial and temporal scales. These changes are driven by the seasonal and interannual climate variations, long-term climatic shifts, and both natural and human disturbance. While the biophysical variables described above are the most prominently noted as drivers of spatial heterogeneity other social/discrete variables are responsible for localized changes on the landscape. In this study we look to incorporate both the regional and local scale drivers of change by including variables such as: soil type, population, elevation, protected areas, country boundaries, and distance / buffer measures related to water and development infrastructure. Table 1 denotes the social/discrete data used in this analysis, as well as the data source, and Fig 2 represents the spatial agreement / disagreement between the regional DFA model predictions and the actual MODIS NDVI data.

**Table 1:** Time series (biophysical) and discrete (mostly social) data used in the DFA and Random Forest portions of the study.

**Data Type**	**Name**	**Description**	**Source**
**Biophysical time series data used to derive DFA model for NDVI_pred_**	Precipitation	Monthly Precipitation	Matsuura and Willmott
	Temperature	Monthly Mean and Max Temperature	Matsuura and Willmott
	Evapotranspiration	Monthly Potential Evapotranspiration	NCEP-DOE Reanalysis II
	Fire	Thermal Anomalies & Fire	MODIS (MOD14A2)
	Soil Moisture	Monthly Soil Moisture	CPC
**Random Forest data: Social / Physical Discrete**	Parks / Protected Areas	National Parks & Conservation Areas	World Database on Protected Areas
	Country	Political Boundaries	ESRI
	Population	Gridded Population data	Afripop
	Soil	Gridded soil type	FAO
	Land Cover Type	Major ecosystem land cover classification	Global Land Project
	Distance to Road	Euclidian distance to tar and unpaved roads	Created
	Distance to Town	Euclidian distance to human settlements	Created
	Distance to Water	Euclidian distance to permanent waterway or body	Created
	Within 1 km of a Settlement	Binary variable related to distance to settlements	Created
	Within 90m of a Road	Binary variable based on buffering roads 90m	Created
	Within 90m of a River	Binary variable based on buffering rivers 90m	Created
	Inundated Areas	Wetlands, Swamps, or any region that could hold water during floods	DIVA-GIS
	Elevation	DEM	SRTM
	Slope	DEM Derived, change of elevation for each DEM cell	SRTM
	Aspect	DEM Derived, downward direction of max. change	SRTM

While the previous DFA analysis captured major drivers of landscape change across this region, in this study we further defined drivers of spatial variability by evaluating where the regional DFA model performed well over time and space (i.e NDVI_obs_ = NDVI_pred_), and where the DFA model failed (i.e. NDVI_obs_ ≠NDVI_pred_). We then examined discrete physical variables (e.g. elevation, soil type, inundated areas, land cover type) and a suite of political or social variables (e.g. parks, distance from roads and towns, population, country) as explanatory drivers of the under or overprediction of NDVI using a random forest (RF) classification approach, but differently from typical applications of the algorithm. The under prediction of NDVI by the DFA analysis means that the landscape is typically more biologically productive than represented in the model; whereas, the over prediction of NDVI is associated consistently, with a less productive landscape than predicted. Combined, the DFA and RF methodologies enable us to highlight drivers of change from time series and discrete variables driven by natural or human impacts.

### Determining differences in the NDVI_pred_ and the NDVI_obs_

To identify trends in under- and over- model prediction we first differenced every pixel of NDVI_obs_ (pixel-level) from the monthly NDVI_pred_ (polygon-level). Following the differencing of NDVI_obs_ and NDVI_pred_ the scale of analysis is at the pixel scale for all subsequent analysis. We detected trends in the difference between NDVI_obs_ and NDVI_pred_, at the pixel level, utilizing the seasonal Mann-Kendall’s *τ* test (SMK; Fig 2A, Supplemental data). This non-parametric test is widely used to evaluate the significance of trends in time series such as precipitation, water quality and other environmental factors because it is robust against non-normality, missing values, seasonality, and serial dependence [30,31]. If τ is greater than zero and statistically significant, there is an increasing trend; if τ is less than zero and statistically significant, there is a decreasing trend. The extent of the trend can be represented by the median of Sen’s slopes for all months, which can be expressed as:

$$\beta_{i}=median(\frac{x_{ij}-x_{ik}}{j-k})$$ (1)

$$\beta =median(\beta_{1},\beta_{2},\beta_{3}\ldots \beta_{12})$$ (2)

where *β*_*i*_ is the Sen’s slope, the median slope joining all pairs of observations, for month *i*, and *β* is the grand slope for the time series. The positive value indicates an increasing trend, and vice versa. The trend surfaces illustrate where, with statistical significance the DFA under-predicted (productivity / greenness higher than predicted) or over-predicted NDVI (productivity / greenness lower than predicted; Fig 2). Areas not identified as over or under predicted in the SMK trend surface are areas where the regional DFA predicted the finer scale NDVI pixel values (i.e. productivity / greening trends).

### Random forest analysis

A Random Forest (RF) analysis was run on the SMK trend surface. Random Forest is an ensemble learning technique developed by Breiman [32], and like decision trees, looks to model response variables (e.g. land cover type, percentage tree cover) from a set of covariates by generating a classification tree [33,34]. Where decision tree classifiers generate one tree, RF generates hundreds of trees (forests), which are aggregated to compute a classification. The RF response is determined by evaluating the response of all the trees, and for classification, the class that predicted most is the class assigned for that object. For example, if 500 trees are grown and 400 of them predict a pixel to be woodland and 100 predict it to be grassland, the predicted outcome for that pixel is woodland [34]. Each tree is grown using a different bootstrap sample *X*_*i*_ of the original data set *X* and the nodes are split using the best predictive variables among a subset of randomly selective predictor variables [35,36].

To decrease the time it takes to construct trees, RF tests subsets of the predictor variables for each decision branch (node). Roughly 2/3 of the reference data are sampled with replacement to build each tree, while 1/3 of the reference data are withheld from tree construction (called “out-of-bag”, or OOB error) [34]. OOB is used to get a running unbiased estimate of the classification error as trees are added to the forest. Other RF outputs essential to interpretation of the classification include the variable importance plots, which explain how important each variable is in classifying the data. Related to the variable importance plots are two metrics, mean decrease in accuracy and the Gini coefficient, which are related to OOB and node impurity. The mean decrease in accuracy is determined using the out of bag error calculation [32]. Specifically, the more the accuracy of the RF decreases due to the exclusion of a single variable, the more important said variable is deemed; therefore, variables with large decreases in accuracy are more essential to the RF and resulting classification of the data [32]. The Gini coefficient, often referred to as the mean decrease in Gini, is associated with node impurity. Overall, the mean decrease in Gini is a measure of how each variable contributes to the homogeny of the nodes and leaves in the resulting forest. Each time a split of a node in a tree is made on a variable, the Gini impurity criterion for the two new nodes is less than that of the original parent node [32]. Adding up the Gini value for each individual variable over all trees in the forest gives a variable importance metric [32].

Utilizing the trend surfaces derived from the Kendall’s correlation coefficient (*τ*) RF classification technique (R package “ModelMap” and “randomForest”) was used to further define variables influencing the composition of NDVI over and under prediction [35,37]. Inputs to the Random Forest classifier (Table 1, Fig 2) include discrete variables ranging from elevation to country boundaries. Data preprocessing of these predictor variables varied according to the dataset. Buffers were placed around roads and waterways as structures have an impact on the vegetation surrounding them, not just those directly in contact. Additionally, using a Digital Elevation Model (DEM), measures of slope and aspect were calculated in ArcGIS 10.1 [38]. Lastly, Euclidian distance measurements were taken for the settlements, roads, and waterways data layers. To perform the Random Forest methodology, in ArcGIS 10.1 a random sample of points was distributed across the study site, in regions that were over or under-predicted with statistical significance [38]. In this study, we use the Random Forest approach to determine what social and physical discrete variables may be driving the vegetation biomass trends, which were poorly predicted by the regional DFA.

## Results and discussion

### Predicted NDVI values—Dynamic factor analysis

Understanding the environmental factors driving NDVI is key to understanding how projected climate and land-use change will affect local and regional vegetation. The DFA results support the long understood main drivers of spatial heterogeneity: soil moisture, precipitation, and fire, but also illustrated the little mentioned but significant drivers of temperature and potential evapotranspiration on NDVI in savanna systems [16]. Using this model we could predict the NDVI values at a pixel level and at a monthly time step for 2001–2010. Fig 2C illustrates those regions where this model fully explains the monthly time-series of NDVI. The correct predictions of 57.18% of the landscape by the DFA analysis alone shows how savanna landscapes are dominated by biophysical—mainly climate—drivers. However, this leaves 42.82% of the landscape unexplained.

### Areas of change–where NDVI_pred_ ≠ NDVI_obs_

There are areas where the model under-predicted (27.46% of the landscape) and over-predicted (15.35% of the landscape) NDVI (Fig 2C), meaning that across the study area 27.46% of the landscape had higher productivity and 15.35% had lower productivity than the regional DFA model predicted. The seasonal Kendall test was applied to detect the trends in the difference between the observed (monthly NDVI_obs_ pixel value) and the predicted NDVI (monthly NDVI_pred_ polygon values). Fig 2A and 2B illustrate the patterns in the trend analysis. Positive values in both figures indicate increasing trends, where the actual values of NDVI are greater than that predicted by the DFA (under-prediction). Negative values in both figures illustrate decreasing trends, areas less green (lower NDVI) than predicted by the DFA (over prediction).

A distinct spatial pattern emerged from the trend analysis. In the southern portion of the study area, the DFA tended to under-predict NDVI, meaning another variable or variables were driving patterns of vegetation productivity. The exception to the pattern is around the Okavango Delta, in the southernmost portion of the study area. In this wetland region, the DFA consistently over-predicted NDVI and therefore vegetation abundance. This over-prediction of NDVI is most likely due to seasonal inundation across the Okavango Delta, due to water having low NDVI values. In the northern region of the study area, the DFA over predicted NDVI, i.e. the model predicts the landscape to be greener than it actually is, meaning some other variable or variables are causing lower NDVI values, perhaps related to heavier human impact in woodland landscapes.

### Random Forest–drivers of over and under predicted NDVI

The DFA model accurately predicted the monthly NDVI values for 2001–2010 for more than half of the landscape. With the RF analysis, we can accurately explain the drivers of 75.2% of the under and over-predicted regions. Therefore, with these combined methods we determined the drivers (both social and ecological) of NDVI across this landscape from 2001–2010 for 89.38% of this highly heterogeneous savanna ecosystem.

**Fig 3:**
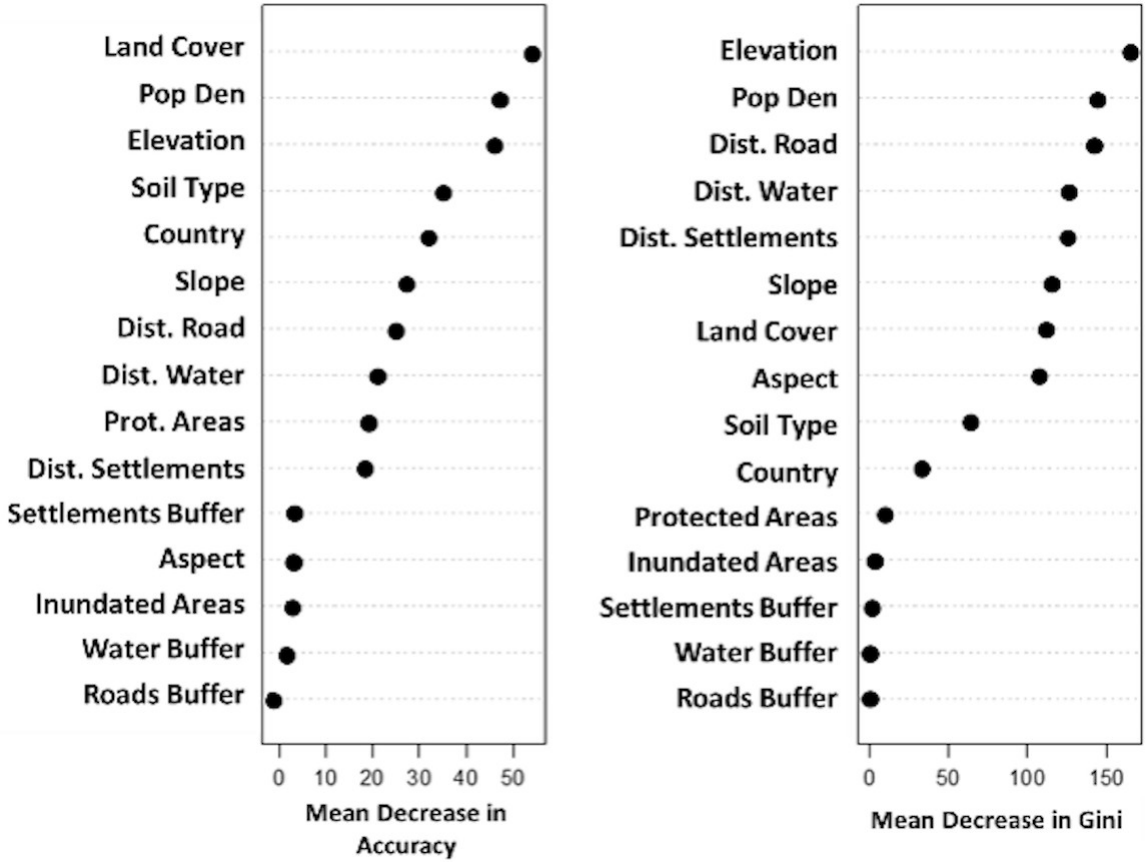
Overall variable importance for both under and over-predicted areas from the RF model.

**Fig 4:**
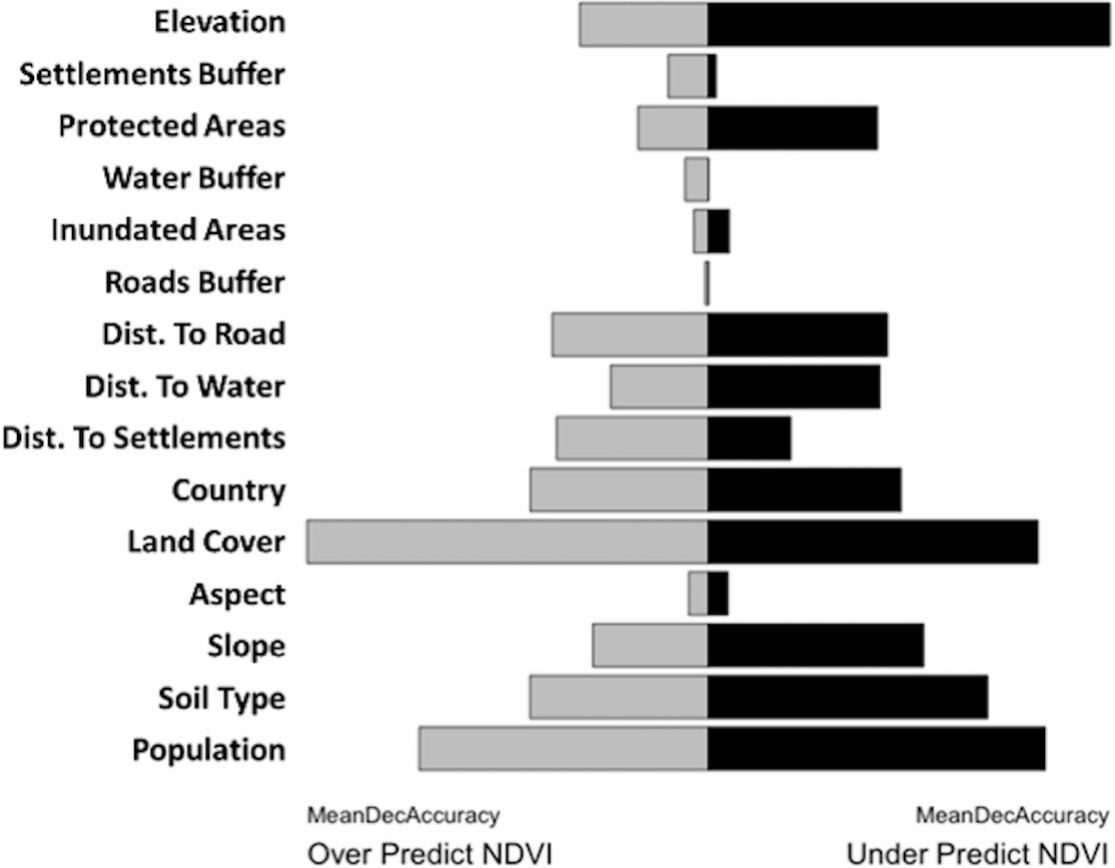
Comparison of the proportional importance of covariates in under and over predicting NDVI based on the DFA model, from the RF analysis.

The strength of the RF model, built based on the Kendall trend analysis, had a very low OOB error rate, meaning that the resulting model was strong based on the numerous classification trees built with the RF bootstrapping technique. Overall, the RF analysis showed that numerous static social and environmental variables could further our understanding of the amount and pattern of NDVI. The combined model showed that elevation, land cover type, population, soil type, country, protection status, and distance measures were influential in the pattern of NDVI (Fig 3). Across these regions, the main drivers of landscape change are typically associated with environmental variables such as soil moisture [16] and not social or environmental variables, such as population or elevation. The cumulative RF model illustrates the impact of human use of the landscape, and elucidates understudied drivers of landscape pattern and change. Interestingly, different variables were found to be important in driving trends in the under- and over-predicted areas, and when the same variable was significant, the strength of the relationship differed between under and over predicted areas (Fig 4). For example, elevation was important for both under and over predicted areas, but was more significant in the under predicted NDVI pixels.

**Fig 5:**
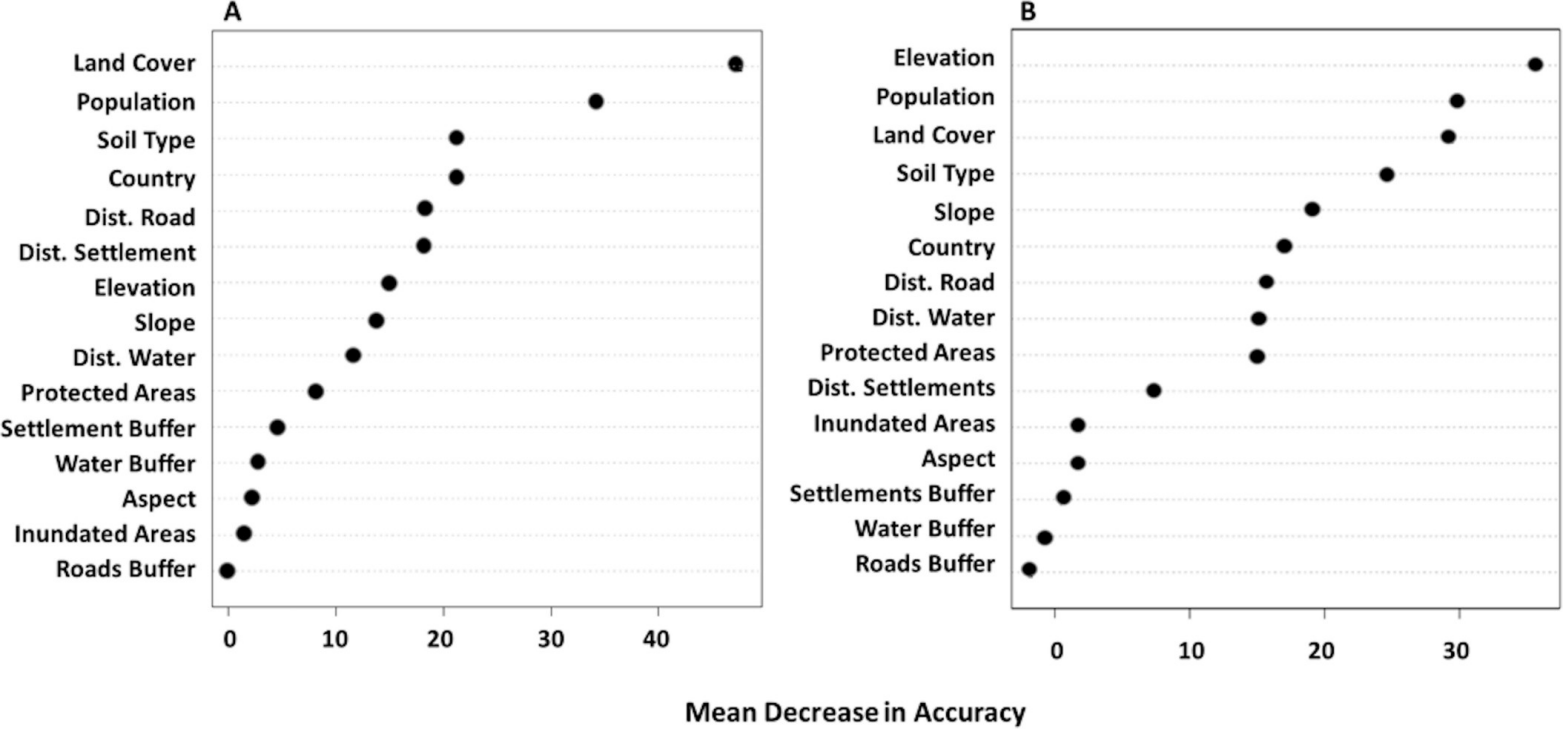
Variable importance, measured by mean decrease in accuracy, for (A) the DFA over-predicted regions and (B) the DFA under-predicted region from the RF model.

To examine variable importance in over- and under predicted areas, importance was plotted and histograms of covariate trends were constructed. In general, the northern portion of the study area is more degraded. In degraded areas (in which the model over-predicted NDVI), the most important variables were land cover type, population, soil type, and measures of human influence including distance to settlements and roads, as illustrated by the mean decrease in accuracy resulting from the Random Forest classification (Fig 5A). In the regions where NDVI was over-predicted, humans are the main drivers of landscape change.

In regions where the DFA under-predicted NDVI a greener landscape occurs than was predicted. The variables associated with this greening pattern include elevation, soil type, distance to settlement, seasonally inundated areas, protection status, and population (Fig 5B). Some of these variables are strikingly similar to those driving the landscape patterns in over-predicted areas, but there are important differences.

**Fig 6:**
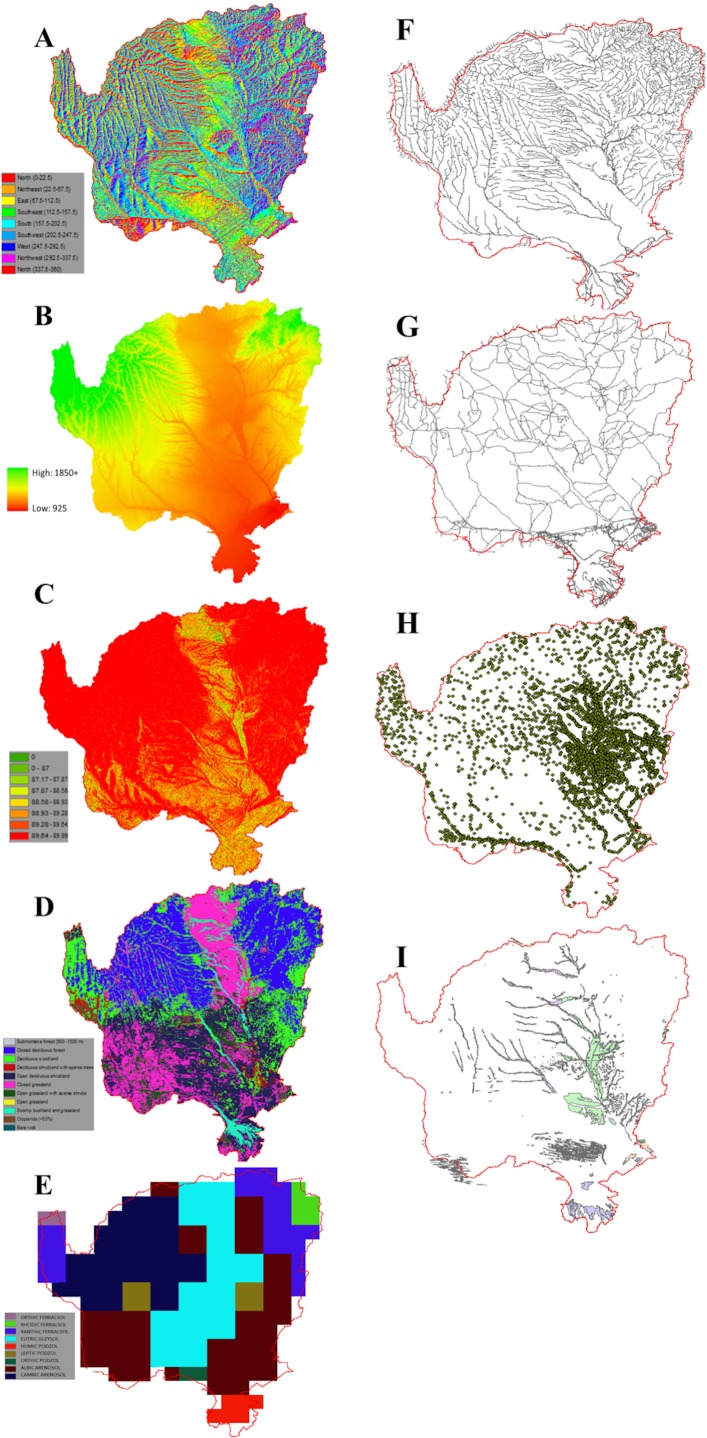
Random Forest Covariates. (A) Aspect, (B) Elevation, (C) Slope, (D) Land Cover, (E) Soil Type, (F) Waterways / Rivers, (G) Roads, (H) Settlements / Cattle Posts, and (I) Inundated Area.

**Fig 7:**
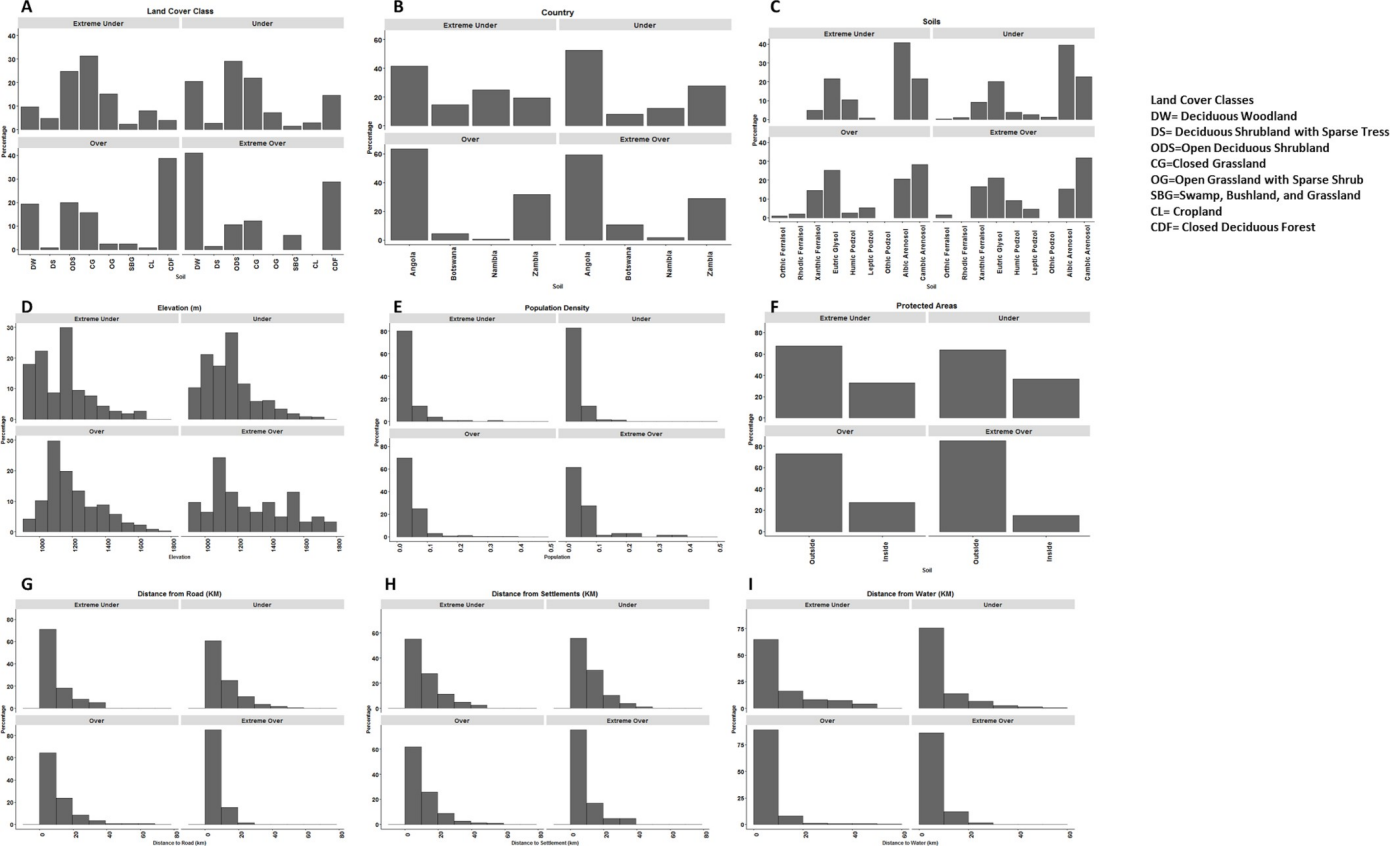
Histograms of the covariates used in the random forest analysis, illustrating the distribution of values associated with under and over predicted NDVI values from the DFA.

Figs 6 and 7 illustrate the human and environmental covariates and their distribution of values in relation to the dependent variable (under or over predicted NDVI). The histograms (Fig 7) have been broken up into classes of extremely over / under predicted and significant over / under predicted based on Kendall τ (SMK) values. Land cover was important in both the under and over predicted pixels. The common land cover types in the over-predicted regions were: closed deciduous forest, deciduous woodlands, and bushlands. In the case of the deciduous woodlands and the bushlands (aka swamp, bushland, and grassland) there were extreme over predictions (~33% of the pixels). All of these land cover classes would typically be associated with higher NDVI, but given the pattern of human use, these values of NDVI were lower (i.e. while there is dense vegetation in this region, given the environmental conditions, the NDVI is not as high as it could be). The land cover conversions that have been seen in this region illustrate people’s dependence on the landscape, including for agriculture and grazing lands. Land cover classes associated with under prediction of NDVI include: deciduous shrubland, open deciduous shrubland, closed grassland, and cropland. The under prediction of cropland illustrates that productivity is higher in these croplands than would be predicted for natural vegetation. This makes sense as these annual crops do have high growth compared to the surrounding vegetation. Closed grasslands had a high proportion of extremely under predicted (34%) and under predicted (22%); whereas only 17% were over predicted and 13% extremely over predicted indicating that savanna grasslands are highly productive systems compared to shrublands. Overall, deciduous shrublands make up a small percentage of the pixels studied, but they were slightly more often under predicted. Lastly, open deciduous shrublands were one of the more prevalent land cover types in the study area. A small proportion of the areas were over predicted but approximately 25% were extremely under predicted and 28% were somewhat under predicted.

As previously mentioned, there is a striking difference in under versus over predicted NDVI pixels progressing north to south across the study area (Fig 2). This pattern is highlighted by two variables: country boundary and elevation (Fig 6). The country boundary histograms (Fig 7B) illustrate the north to south pattern under versus over predicted. Angola covers the largest proportion of the study area and has large percentages of both under and over predicted pixels, though there was a higher probability of over prediction. Progressing southward, the pattern shifts and there is a high probability of under-predicting. An example of this occurs in Namibia and Botswana, where over-prediction occurs in less than 15% of the pixels but under predicted NDVI much more often (Botswana upwards of 18%; Namibia upwards of 27%). From north to south there is a decrease in elevation (Fig 6). At higher elevations (greater than 1500 m), in the north, the DFA model is much more likely to over predict NDVI. As we move down the elevational gradient this pattern inverts. From 1200–1500 m in elevation there is an almost equal mix of under and over predicted NDVI pixels; whereas, at elevations less than 1200 m NDVI is more likely to be under predicted.

Each of our distance metrics (distance to roads, settlements and water) played a role in the pattern of under and over prediction across the study area. The distance to road and settlement measures highlight the human footprint on the environment. With both variables the closer to infrastructure the more likely we are to over predict NDVI (Fig 7G and 7H) which illustrates the human use of the landscape resulting in lower NDVI due to more built structures and intense uses. Distance to water was, surprisingly, the least significant of the distance metrics (Fig 7I). To see the difference between the under and over predicted pixels in terms of distance to water you really have to compare all of the histograms together, because the differences are minor. In the under predicted areas we see there is a greater proportion of pixels away from water than with the over predicted.

Within protected areas (PAs) there was a tendency to under predict NDVI with the DFA model, but outside PAs was roughly evenly split between under and over prediction. This highlights the success of parks and protected areas in conserving vegetation cover across this landscape and is an important finding for park management.

Not all variables put into the random forest model were significant. However, the results from these variables can contextualize the complex human-environment interactions across the region. For instance, seasonally inundated areas (essentially seasonal wetlands) can sometimes have high levels of productivity. While not very important in the RF model, we found that inside these inundated areas there was a tendency to under-predict NDVI. This could result from a lag between precipitation and changing water levels, and may explain under-prediction by the DFA model.

The use of this combined dynamic factor random forest analysis (DFRFA) allowed for a more complete understanding of both the environmental and social drivers of vegetation cover in this landscape. Under-prediction of NDVI was found at lower elevations, sparser human populations, areas with less human interference (e.g. fewer roads, fewer settlements), and was more common in Botswana and Namibia. Over-prediction occurred in areas of higher elevation, and higher human populations. These results may seem obvious, but are useful to demonstrate the importance of variables in over- and under-prediction together in this landscape. It is a positive result for conservation goals to find that protected areas in this landscape appear to be maintaining vegetation cover, exhibiting a clear pattern of under prediction of NDVI based on environmental variables alone.

## Conclusion

This study builds on our understanding of drivers of spatial heterogeneity across a portion of the semi-arid to arid landscape of southern Africa. This study utilizes a two-step coupled modeling procedure (DFRFA) to analyze the drivers of NDVI, including time series variables (e.g. climate, fire) and static physical and human variables (e.g. distance to roads, water, settlements, soil composition, land cover). The regional DFA was used to generate a model of the drivers of a time series of monthly NDVI data. Then, RF was used to explain the remaining finer scale variance in the landscape that the DFA did not capture. This combined methodology enables us to understand drivers of landscape change beyond traditional statistical methods (regression) and more robustly than the DFA alone. Combined, the DFRFA methodology explained just over 89% of the variance in NDVI across the study area. These tools, used together, provide for an interesting combination–in this case allowing for discrete and continuous drivers to be understood. Furthermore, the combined use of time series and continuous variables enables us to highlight and analyze the differential impacts of biophysical and human drivers of landscape pattern / change. Such a methodological approach was developed at the intersection of Land Change Science, Remote Sensing, and Environmental Modeling / Engineering, highlighting the importance of interdisciplinary research in global environmental change research.

Numerous studies have noted the utility and high level of accuracy associated with RF application [36,39], and there has been a large increase in publications in recent years using RF methodology. [32] showed that RFs do not over fit and there is no need to prune trees [34]. This non-parametric approach is ideal for remote sensing applications as RF implementation produces accuracy, variable importance, and outlier assessments. Decision tree classifiers have an advantage over more traditional classifiers, like maximum likelihood, in that there are no assumptions about data distributions (e.g., normality), they can handle data with different scales, and can adapt to noise and non-linear relationships inherent in remote sensing data [40]. We used RF to explain two surfaces–one of model NDVI over prediction and a second of model NDVI under prediction across our landscape. This novel use of a RF approach complemented the time series based analysis with discrete social and physical drivers, thus providing a higher level of explanation than could be determined by each method separately.

Both methodological approaches showed a distinct north to south pattern in drivers of spatial heterogeneity. Previous research [16] highlighted the importance in the spatial distribution of precipitation, soil moisture, and fire on NDVI. Additionally, this paper pointed out the overlooked or understudied effect of temperature and potential evapotranspiration (PET), especially in regions with mean annual precipitation greater than or equal to 950 mm. This research addresses smaller scale roles of discrete variables across the landscape. In over-predicted areas, humans are the main driver of landscape pattern and change, as shown by the importance of population and land cover type as variables in the RF. In the north, there are higher proportions of deciduous woodlands, closed deciduous forest, and bushlands in over-predicted pixels. These areas have a higher proportion of human utilized landscape, leading to decreased NDVI. Conversely in regions where the DFA under-predicted (greener landscape) the variables with the most explanatory power include elevation, soil, population, and land cover. Some of these variables are similar to those driving landscape patterns in over-predicted areas, but there are important differences, as shown in Fig 7. In the southern portion of the study area, under predicted NDVI pixels were common amongst the deciduous shrublands, open deciduous shrublands, and closed grasslands. The RF analysis revealed that NDVI under-predicted by the DFA occurred in low elevations, especially across Botswana and Namibia. These under-predicted areas had lower human impact on the environment with sparser populations and greater distances to roads and settlements.

A limitation of this study is the lack of data on animals available to this analysis–both wildlife and livestock, aside from cattle post locations. Adding a livestock or wildlife variable might improve predictions where NDVI is over-predicted, as impacts of herbivory are significant across the landscape. However, with almost 90% of NDVI variance explained in this landscape, our combined DFRFA methodology is a significant advance in the study of these systems, and a strong explanatory tool for management. Savanna systems need advanced analysis tools, like DFRFA, to parse out drivers of change in spatially and temporally complex systems. Such tools are useful for management where complex environmental drivers are embedded within landscapes subject to discrete social, political, and economic boundaries. Understanding the interplay of these difference variables is key to their management and monitoring.

## Supporting information

S1 File Link to the data used in the method “seasonal Mann-Kendall’s *τ* test” to detect trends in the difference between NDVI_obs_ and NDVI_pred_, at the pixel level. (ZIP)
